# Protein Synthesis Inhibitors Did Not Interfere with Long-Term Depression Induced either Electrically in Juvenile Rats or Chemically in Middle-Aged Rats

**DOI:** 10.1371/journal.pone.0161270

**Published:** 2016-08-12

**Authors:** Abdul-Karim Abbas

**Affiliations:** Institute of Neuroscience and Physiology, University of Gothenburg, Box 432, SE-40530, Gothenburg, Sweden; Technion Israel Institute of Technology, ISRAEL

## Abstract

In testing the hypothesis that long-term potentiation (LTP) maintenance depends on triggered protein synthesis, we found no effect of protein synthesis inhibitors (PSIs) on LTP stabilization. Similarly, some studies reported a lack of effect of PSIs on long-term depression (LTD); the lack of effect on LTD has been suggested to be resulting from the short time recordings. If this proposal were true, LTD might exhibit sensitivity to PSIs when the recording intervals were enough long. We firstly induced LTD by a standard protocol involving low frequency stimulation, which is suitable for eliciting NMDAR-LTD in CA1 area of hippocampal slices obtained from juvenile Sprague-Dawley rats. This LTD was persistent for intervals in range of 8–10 h. Treating slices with anisomycin, however, did not interfere with the magnitude and persistence of this form of LTD. The failure of anisomycin to block synaptic-LTD might be relied on the age of animal, the type of protein synthesis inhibitors and/or the inducing protocol. To verify whether those variables altogether were determinant, NMDA or DHPG was used to chemically elicit LTD recorded up to 10 h on hippocampal slices obtained from middle-aged rats. In either form of LTD, cycloheximide did not interfere with LTD stabilization. Furthermore, DHPG application did show an increase in the global protein synthesis as assayed by radiolabeled methodology indicating that though triggered protein synthesis can occur but not necessarily required for LTD expression. The findings confirm that stabilized LTD in either juvenile, or middle-aged rats can be independent of triggered protein synthesis. Although the processes responsible for the independence of LTD stabilization on the triggered protein synthesis are not yet defined, these findings raise the possibility that *de novo* protein synthesis is not universally necessary.

## Introduction

Activity-dependent long-term changes in synaptic function, i.e. synaptic plasticity, at glutamatergic synapses are currently a prevalent model used to investigate the cellular basis of learning, memory and behavioral adaptation [1,2]. The two major forms of synaptic plasticity are long-term potentiation (LTP) and long-term depression (LTD). LTD is predominantly mediated by activation of *N*-methyl-D-aspartate glutamate receptors (NMDAR-LTD) or metabotropic glutamate receptors (mGluR-LTD) [3] at hippocampal CA3:CA1 synapses. The durability of LTP and LTD *in vitro* preparations [4,5] is one of the main attributes of LTP and LTD supporting the relevance of synaptic plasticity to learning and memory [6,7]. In analogy to the temporal dissection of memories, LTP, and to a lesser extent LTD, are widely-believed to be divided into two, or more, temporal phases that are distinguished mechanistically, i.e. an early phase (E-LTP/E-LTD), which is presumed to be dependent on posttranslational modifications, and a late phase (L-LTP/L-LTD), which is considered to be induced by *de novo* protein synthesis/mRNA transcription [8,9]. Several studies have shown that the effect of protein synthesis inhibitors (PSIs) on LTD was immediate [10–12], suggesting that, under some conditions, LTD induction is dependent on protein synthesis. Other studies observed a lack of effect of PSI on LTD under specific conditions. For example, Nosyreva and Huber [13] showed that the induction of mGluR-LTD is protein synthesis-independent in hippocampal slices obtained from neonatal but not adolescent animals. However, as the recording intervals reported in that study were relatively short, there remains plausible that the short recording intervals did not allow observing an effect of the interruption in adult rodent, i.e. it may be ‘an issue of detectability’ [14]. If this argument were correct, given that *in vitro* LTD has been usually recorded for no longer than 1–4 h [14], recording LTD for several hours would provide sufficient interval to detect any effect of PSI.

Prolonged Low-frequency stimulation at 1 Hz frequency is a conventional paradigm used to typically induce NMDAR-dependent homosynaptic LTD (LFS-LTD) in juvenile and young adult rodents [10,15–17]. On the other hand, hippocampal NMDAR-LTD and mGluR-LTD can be induced by bath application of NMDA and agonists of group I mGluRs, respectively [1]. Although LTDs induced by these agonists diverge many mechanistic aspects from the LFS-LTD [1,18,19], they occlude further induction of electrical LTDs and thus share them some underlying mechanisms of expression [3,20]. Moreover, chemical-LTD has the advantage of massive synaptic depression [21], which therefore maximizes the probability of detecting biochemical changes [22] and, in consequence, their sensitivity to interruption [23].

Previous studies have shown that generally LTD can be readily induced in acute hippocampal slices prepared from juvenile [17,20] or from adult rodents under specific conditions [17,24], but it is difficult to induce LTD in slices obtained from middle-aged or senescent rodents and, when observed [12,24,25], the recording intervals rarely showed LTD recorded for more than 4 h [12]. However, genetic knockout associated with enhancement of protein synthesis in old mice was associated with enhancement of (*RS*)-3,5-dihydroxyphenylglycine (DHPG)-induced LTD, implying that the difficulty to induce LTD in wild-type old animals may be due to a physiological suppression of protein synthesis [26]. In addition, LTD in middle-aged rodents, primarily induced through activation of mGluRs, was shown to result in protein translation [23,25,26]. Consistently, mice lacking the translational suppressor fragile mental retardation (FMR) protein exhibited enhanced mGluR-LTD [13]. The role of protein synthesis in NMDAR-LTD is even more cryptic than mGluR-LTD. In the CA1 region, the data show that PSIs interfere with LTD induced by NMDAR activation [9], or that PSIs interfere only when mGluRs activation was conjunct [14]. Although no study yet, up to our knowledge, has investigated the role of protein synthesis in the NMDA-induced LTD, it has been shown that NMDA addition before mGluR stimulation markedly depressed the polyribosomal loading suggesting negative contribution of the NMDARs in protein translation [27].

We have previously shown that LTP in CA1 area of rat hippocampal slices from juvenile rats were stabilized for several hours beyond the presumed “E-LTP” time course under condition of protein synthesis inhibition [28]. The present experiment tests whether these findings also apply to electrically- and chemically-induced LTD at the Schaffer collateral-CA1 synapses in juvenile and middle-aged hippocampal slices, respectively. The rationales for using different age groups and different induction protocols include the following facts: firstly, we aimed to assess whether LTD exhibit similar or different response to PSIs to that shown in LTP study performed on juvenile rats. Secondly, we addressed the issue whether age difference has any determinant role on the response of LTD to PSIs. Thirdly, by using these comparative ages/protocols we tried to find which protocol(s) may provide a durable LTD, the case that would be suitable for detecting temporal phases, and lastly, middle-age rats might be good choice to assess the requirement of mGluR-LTD for protein synthesis as shown by Nosyreva and Huber [13].

## Materials and Methods

### Animals and animal care

The experiments in this study were carried out in accordance with the European Communities Council Directive of September 22nd, 2010 (2010/63/EU) for care of laboratory animals and after approval of the local ethics committee (University of Gothenburg). Albino rats (strain Sprague–Dawley) were obtained from Charles River Laboratories (Germany). Two age groups, male and female juvenile between 14 and 24 days of age, and male middle-aged between 40 and 48 weeks of age were used. Prior to the experiment, the rats were group-housed and maintained on a 12 h dark/light cycle and had access to food pellets and water *ad libitum*.

### Electrophysiological recording

Using deep anesthesia (isoflurane, Baxter Medical AB, Sweden), the rat was decapitated between 13.00 and 14.00 p.m. to prevent variations caused by circadian rhythms or nonspecific stressors [29]. This type of anesthesia is less likely to interfere with the electrical (e.g. decreasing the EPSP size) or biochemical (e.g. decreasing the brain protein synthesis) responses as has been reported with other types of anesthetized animals [30]. Despite the fact that middle-aged rats have thicker skulls and tougher connective tissue than younger rats, every effort was undertaken to avoid delay during brain extraction and/or dissection. The whole brain was rapidly removed and immersed in ice-cold dissection artificial cerebro-spinal fluid (aCSF) composed of (in mM): 119 NaCl, 2.5 KCl, 2.0 CaCl_2_, 2.0 MgCl_2_, 26 NaHCO_3_, 1.0 NaH_2_PO_4_, and 10 D-glucose) equilibrated with 95% O_2_, 5% CO_2_, the pH set at ≈ 7.4. This solution contains less Ca^2+^ and higher Mg^2+^ than incubation aCSF (see below) to promote neuronal survival during the slicing procedure by reducing excitotoxicity [31]. The cerebellum was removed and a cut was made to divide the two cerebral hemispheres. The hippocampus of one or both sides was dissected out and 400 μm thick transverse slices were prepared by a tissue chopper at 30° of the longitudinal axis. The mid third of the hippocampus was chosen for the slice cutting. This was based on the fact that anatomical, behavioral, electrophysiological, and pharmacological division of work has been reported to occur at septal (rostral) vs. temporal level of the hippocampus [32–34]. Following preincubation for at least 90 min at room temperature in oxygenated aCSF, the slices were individually transferred as needed to a submersion-type recording chamber, placed between nylon net and a set of parallel nylon threads attached to a U-shaped platinum wire to prevent their movement. The chamber consisted of circular well of a low volume (1–2 ml) perfused continuously, using a peristaltic pump (Ismatec, Labinett Lab AB, Sweden), with warm (31°C) oxygenated aCSF (similar to the dissection solution, but with CaCl_2_ and MgCl_2_ concentrations at either 2.5 and 1.3 or 1.5 and 1.3, respectively) at a flow rate of 1.5–2 ml/min. The chambers and their inlets and outlets were routinely sterilized using penicillin-streptomycin solution at final concentration of 500 units for penicillin and 0.05 mg for streptomycin per ml before running each experiment. To avoid contamination of the antibiotics, the chambers were washed with distilled water for at least 1 h.

Each chamber is provided with a recording electrode, made by a glass micropipette filled with 1 M NaCl (R = 3–5 Mῼ) to register presynaptic fiber volleys followed by field excitatory postsynaptic potentials (fEPSPs), and two monopolar tungsten stimulating electrodes (0.1 Mῼ; World Precision Instrument, Inc., Sarasota, FL, USA) positioned in the middle of the CA1 stratum radiatum at equal distance on sides of the recording electrode (100–200 μm) for experiments designed for delivery of low- or high-frequency stimulation whereby one stimulating electrode served to stimulate a control input. Alternatively, the chamber was provided with one stimulating electrode when chemical LTD was performed. Electrode positioning was controlled by a mechanical 3D micromanipulator operated by hand and performed under visual guidance using an upright “Nikon” stereomicroscope equipped with a 3.5x objective and a 20x ocular. The recording electrodes were pulled from microfiber capillary tubing (o.d. 1.5 mm, i.d. 0.86 mm, Warner Instruments, LLC, Hamden, CT, USA) and the position of the stimulating electrode was alternated between the CA3 and subicular side of the recording electrode in different experiments [35]. Pulses, delivered as 100 μs negative constant current (10–50 μA) using a programmable pulse generator, were repeated alternately to the two pathways at a rate of once every 40 s with stimuli to the two pathways separated by 20 s when LFS was used, or once per 20 s for the chemical LTD experiments.

### Pharmacological compounds and drug treatments

The *N*-methyl-D-aspartate (NMDA), the NMDA receptor (NMDAR) specific competitive antagonist D(-)-2-amino5-phosphonopentanoic acid (D-APV), and the group I mGluR agonist (*RS*)-3,5-dihydroxyphenylglycine (DHPG) were obtained from Tocris Cookson (UK) or Ascent Scientific Ltd (UK). Cycloheximide (4-[(2R)-2-[(1S,3S,5S)-3,5-Dimethyl-2-oxocyclohexyl]-2-hydroxyethyl]piperidine-2,6-dione), anisomycin (2-[p-methoxybenzyl]-3,4,pyrrolidinediol-3-acetate), penicillin-streptomycin solution, and dimetylsulfoxide (DMSO) were all purchased from Sigma-Aldrich (St. Louis, MO, USA). [^3^H]leucine was obtained from Amersham, Buckinghamshire, UK. Milli-Q deionized water (Millipore, Bedford, MA, USA) was used in all preparations of buffer solutions. Other chemicals used were all of highest grade commercially available.

The drugs were made up as stock solutions in either double-distilled water (D-APV, NMDA, anisomycin and DHPG) or 99% v/v. DMSO (cycloheximide) at 10-1000-fold their final concentration and stored at -20°C. DMSO at final concentration of 0.01–0.1% was added to the solution of the control group as vehicle to balance any slight effect it might have on LTD. These stocks were diluted in aCSF to achieve their desired final concentrations and were applied by switching the perfusion from control aCSF to drug-containing aCSF.

### LTD induction protocols

Slices that failed to exhibit stabilized fEPSPs within 90 min were rejected, otherwise LTD was induced either electrically by the delivery of LFS which usually involved three trains of LFS (3xLFS), each of 900 pulses at 1 Hz for 15 min (2700 pulses total), to induce a fully saturated LTD [36] or chemically (cLTD) with one of two treatments: 1) 10 min DHPG (100 μM) bath application; 2) 3 min NMDA (20 μM) bath application. These concentrations and durations are extensively reported to induce LTD in acute hippocampal slices [12,22,37–41]. For the DHPG-LTD experiments, D-APV (50 μM) was added to the bath solution for at least 40 min before the addition of DHPG and usually maintained for the entire duration of the experiment (~10 h). The purpose of D-APV addition was to insure isolated mGluR-LTD. It has previously been shown that lowering the extracellular Ca^2+^ concentration enhanced the DHPG-induced LTD in adult animals [42]. Therefore 1.5 mM instead of 2.5 mM Ca^2+^ was used for these experiments, revealing a robust and long-standing LTD. Efforts were undertaken to avoid the tonic effect of the drugs after washout by continuous circulation of the bath solution for at least 10–15 min.

### Data analysis

Signals were amplified, filtered at 4 kHz and transferred to a PC clone computer for on-line and off-line analysis by specially designed electronic equipment (based on an Eagle Instruments multifunction board) and own developed computer software. Off-line data analysis was performed using a combination of Quickbasic (Microsoft) and Igor (Wavemetrics, Inc.) programs. The slope (mV/ms) of dendritic fEPSPs was measured during a 1–2 ms interval positioned just after the presynaptic volley (early AMPA component) and data for each experiment was normalized relative to baseline recording. The amount of LTD was averaged for each time-point following the last LFS train or the drug washout and expressed as percentage of baseline values (calculated from fEPSPs recorded during the last 15 min of baseline values (100%). The 11–16 successive fEPSPs were averaged and stored digitally for off-line measurement of slope. For each measurement, the mean, standard deviation, and standard errors were calculated for all the included experiments. The data are expressed as means ± SEM. As normality assessment (Q–Q plot and Shapiro–Will test) revealed Gaussian distribution of groups, the parametric Student’s *t*-test was conducted to assess the significance of the comparisons between the groups of slices. Values of less than 0.05 were considered to be significant. Cumulative histograms indicated LTD magnitude (mean ± SEM from groups of slices) after delivery of LFS or the application of DHPG or NMDA alone (control LTD) or in the presence of PSI. All statistical analyses were performed using IBM SPSS (Version 22).

Although we have avoided conducting an input/output (I/O) assessment because this might lead to metaplastic changes [43], every effort was spent to adjust stimulus intensity to evoke fEPSP of nearly the same size across experiments and to avoid population spike appearance [12].

### Biochemical assays

The effect of DHPG on protein synthesis in whole slices was measured by [^3^H]leucine incorporation as described previously [28]. Briefly, hippocampal slices from 24-day old rats were maintained under conditions similar to the electrophysiological experiments but devoid of electrical stimulation. Slices were put on a multiwall plastic dish (Corning Incorporated, Corning, NY, USA) and assigned to a DHPG group or a control group in an interleaved manner to minimize inter-slice variability with respect to weight and metabolism. Two slices per group were pre-incubated for 60 min at room temperature with oxygenated aCSF containing 2:2 Ca^2+^/Mg^2+^ concentration, in equivalent to that for the electrophysiological experiments. Subsequently, slices were transferred to a modified 8-well dish wherein aCSF (2.5:1.3 Ca^2+^/Mg^2+^) continuously perfused slices at 31°C S1A S1. After 20 min, slices were treated with either DMSO-aCSF (control) or DHPG/D-APV-aCSF (100 μM/50 μM) for 10 min. Following few minutes of slices washout with warm aCSF, slices were moved to other chambers destined for radioassay. The slices were incubated therefor 50 min in tritiated leucine bath solution (final activity 0.5–1 μCi/ml). Thereafter, the termination phase via rinsing the slices with ice-cold saline and addition of ~1 ml of 5 mM NaOH was performed. After protein purification, incorporation of leucine into trichloroacetic acid (TCA)-perceptible proteins S1B S1 was measured in a scintillation counter (LKB Wallace, 1219 Rackbeta, Finland). Percentage of leucine incorporation produced by DHPG/D-APV was calculated by comparing counts in treated slices with those of control ones.

## Results

### LFS induced a robust and durable LTD in juvenile hippocampal slices

**Fig 1:**
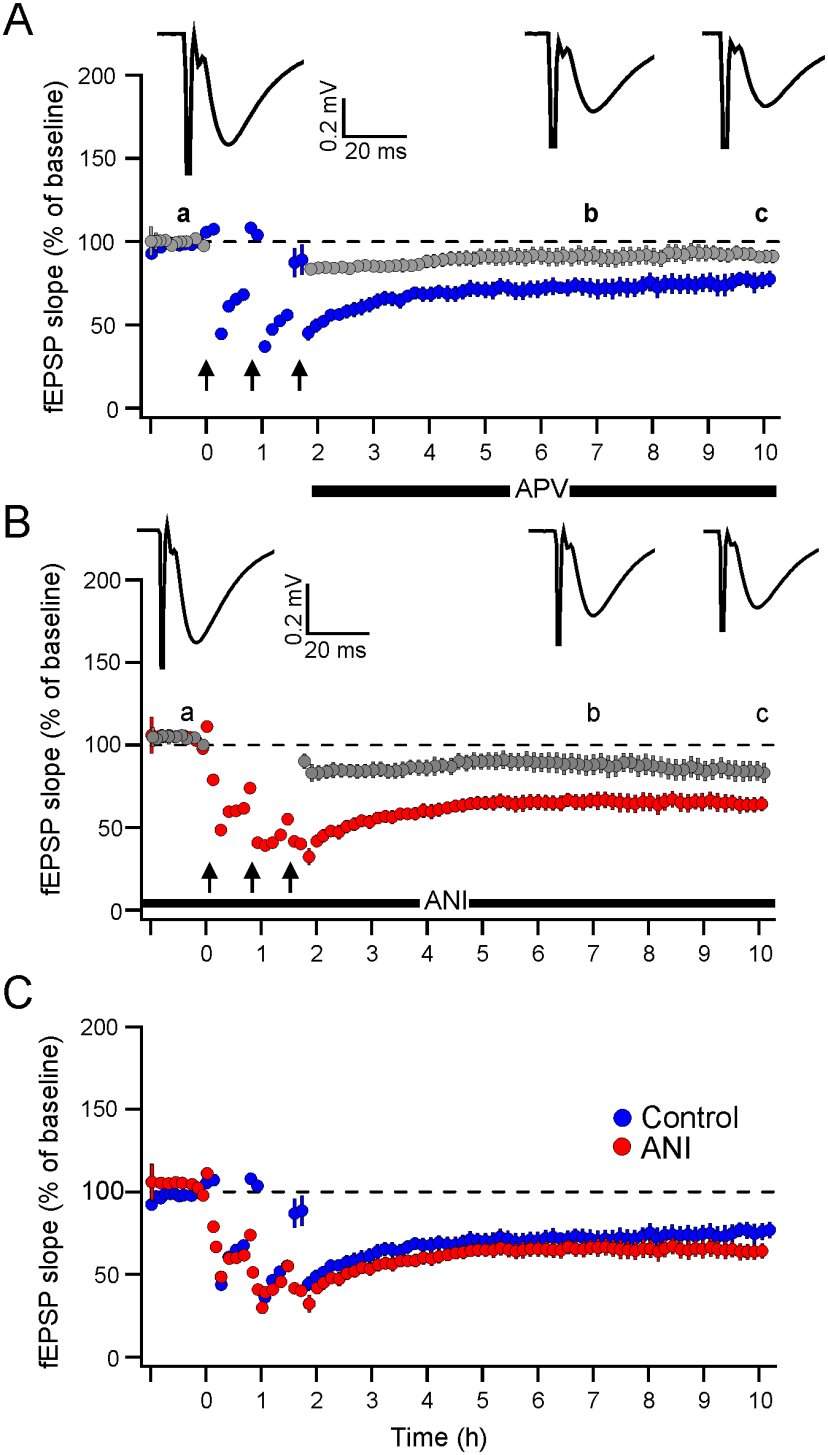
Effect of anisomycin on LFS-LTD. Successive measurements of fEPSP are shown for successive responses in two separate pathways. Each point represents the average of 13 successive responses (~5 min) of individual experiment and averaged of total number of experiment (*n*). (A) Three trains of LFS (arrows) were delivered to one pathway (test pathway). Stimulation of a second, independent pathway (control pathway) was interrupted during the delivery of LFS. D-APV was added to the solution immediately after the end of the last LFS trains (horizontal bar). (B) The same condition as with (A) but 40 μM of anisomycin (ANI) was applied 30 min before the first LFS train and kept in the solution throughout the experiment. (C) Superimposed results shown in A (blue circles) on results depicted in B (red circles) indicating no effect of exposure to anisomycin in terms of magnitude or time-course compared to control LTD. Inset traces in (A) and (B) are averages of 13 responses taken at (a) before LFS, (b) 5 h, and (c) 8 h following the cessation of the last LFS train as indicated in the graphs. Calibration bars: vertical 0.2 mV, horizontal 20 ms.

Three trains of LFS were delivered to one pathway of hippocampal slices obtained from 14–22 days old rats. D-APV was added to the solution immediately after the end of the last LFS trains to block any plausible NMDAR-mediated decay that might be induced by the test stimulation. A robust LTD maintained for up to 8–10 h was observed in control slices (*n* = 5). The LTD amount estimated as percent of baseline values, were 52 ± 4%, 66 ± 4% and 62 ± 4% at 2 h, 4 h and 8 h following the cessation of the last LFS train, respectively (Fig 1A). In the second group of slices (*n* = 5), 40 μM anisomycin was added 30 min before the delivery of LFS and kept in the bath solution throughout the recording time course. However, there was no visible difference in LTD magnitude and duration compared to control LTD (Fig 1B vs. 1A). The LTD amount in the anisomycin-treated slices reached to 55 ± 4%, 69 ± 5%, and 62 ± 6% at the corresponding time points of control LTD. Statistical analysis revealed no significant difference between the groups at any time point (*p* > 0.05; two-sample *t*-test), indicating that anisomycin had no effect on this form of LTD. Fig 1C depicts superimposed control LTD and anisomycin-treated LTD, showing the lack of effect of anisomycin on LTD, on the one hand, and ruling out that anisomycin could alter the synaptic strength on the other hand (cf. [44]).

### NMDA induced a sustained LTD under condition of protein synthesis inhibition

**Fig 2:**
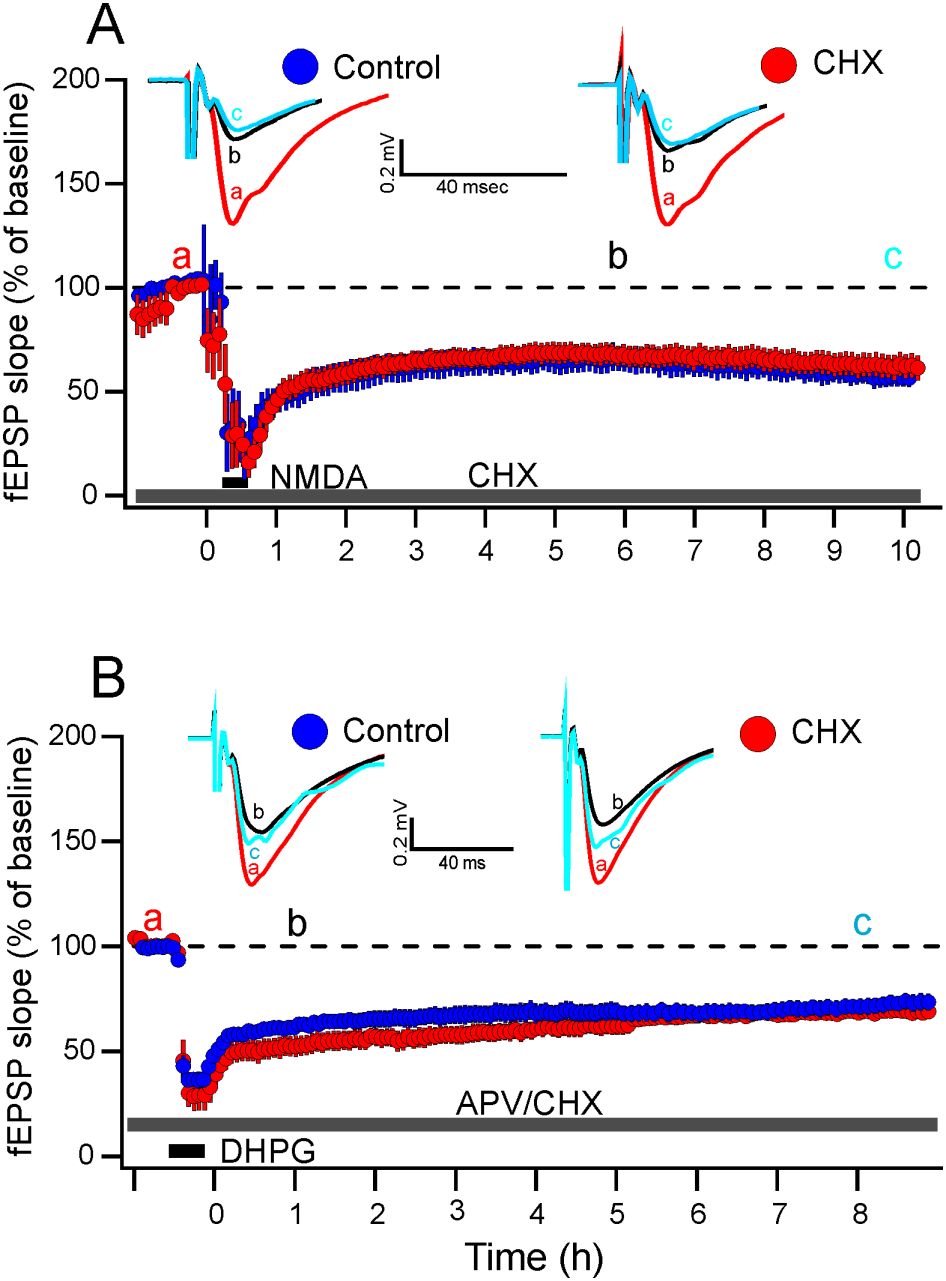
Effect of cycloheximide on NMDA- and DHPG-LTD. Successive measurements of fEPSP are shown for successive responses in one pathway. (A) Pooled data (*n* = 5) from middle-aged rat age group reveals a robust LTD induced chemically by addition of 20 μM NMDA for 3 min (black horizontal bar) after at least 60 min of baseline recording (Control, blue circles). The DMSO was not shown for the lack of space. Superimposing data obtained from slices incubated with 100 μM of cycloheximide (CHX; *n* = 5, grey horizontal bar) on control data reveals no difference in LTD magnitude at any time point. Each fEPSP represents mean (± SEM) of 16 successive responses (~5 min) of *n* experiments. Inset traces are superimposed averages of 16 responses taken at (a) before NMDA addition, (b) 6 h, and (c) 10 h following NMDA addition for control (left) and CHX (right) groups as indicated in the graph. Calibration bars: vertical 0.2 mV, horizontal 40 ms. (B) Pooled data (*n* = 6) from middle-aged rat age group reveals a robust LTD in hippocampal slices induced chemically by addition of 100 μM DHPG for 10 min (black horizontal bar) after at least 60 min of baseline recording (Control, blue circles). D-APV (50 μM) and DMSO (grey bar) were added to the aCSF throughout the time course of recording interval. In this group of experiments the Ca^2+^ concentration in the aCSF was lowered to 1.5 mM in accordance with Watabe et al.’s [42] finding showing that lowering the extracellular Ca^2+^ concentration enhanced the DHPG-induced LTD in adult animals. Superimposing data obtained from slices incubated with 100 μM of cycloheximide (CHX; *n* = 5, grey horizontal bar) on control data reveals no difference in LTD magnitude. Inset traces are superimposed averages of 11 responses taken at (a) before DHPG addition, (b) 1 h, and (c) 8 h following DHPG addition from control (left) and CHX (right) groups as indicated in the graphs. Calibration bars: vertical 0.2 mV, horizontal 40 ms.

It is widely reported that LFS protocols induce readily NMDAR-LTD early in development and in senescent tissue but less effective at inducing LTD in middle-aged animals [16,45]. However, by addition of 20 μM NMDA for 3 min to slices pre-incubated with vehicle DMSO, field excitatory postsynaptic potential (fEPSP) transiently decreased to minimal value but steadily recovered reaching a stabilized phase approximately 3 h after the start of drug washout. Data depicted in Fig 2A (blue circles) reveals depression values on average of 60 ± 6%, 63 ± 7% and 55 ± 3% of the initial baseline level, at 3 h, 6 h and 10 h, respectively (*n* = 5).

By obtaining durable LTD in acute slice preparation we achieved an essential prerequisite for assessment of another PSI on LTD stabilization and, in consequence, for verification the temporal dichotomy of LTD. However, addition of cycloheximide at 100 μM concentration, a value that has been shown to exhibit above 90% protein synthesis inhibition [28], did not change the magnitude or duration of LTD. Measurements at 3 h, 6 h and 10 h yielded values on average of 65 ± 5%, 67 ± 5% and 63 ± 11%, respectively (Fig 2A, red circles; *n* = 5), which are non-significantly different from LTD in cycloheximide-free slices. As shown in Fig 2A, superimposing data from the two groups of experiments discloses the unlikelihood of cycloheximide-induced depression of the synaptic transmission, in accord with our previous findings [46].

### DHPG induced a sustained LTD under condition of protein synthesis inhibition

According to few reports, the independency of 3xLFS-LTD in juvenile rodents [10] on the protein synthesis shown in the present study might not be surprising. Additionally, there is a lack of studies regarding NMDA-LTD and protein synthesis. In contrast, mGluR-LTD is widely-held to be protein synthesis-dependent [10,47]. We then moved to examine whether DHPG can induce LTD that would be stable for as long as that induced by NMDA and if so, whether DHPG-induced LTD is or is not protein synthesis-dependent. Following a rapid reduction in synaptic transmission caused by incubating the slices with DHPG and DMSO, a partial recovery on washout revealed a persistent and stable LTD lasting the duration of over 10 h post-DHPG recording period. As Fig 2B shows, DHPG depressed fEPSP to amounts reaching 68 ± 1% and 66 ± 2%, and 71 ± 2% at 3 h, 6 h, and 8 h following the drug washout, respectively (*n* = 6, blue circles). Of note, D-APV was added to the bath solution throughout the time course of the experiment to insure isolated mGluR-LTD. Although a comparative control group wherein D-APV was omitted was not conducted, the LTD robustness and its persistent characteristic confirm that DHPG-LTD was independent of NMDAR activation, the finding which is consistent with previous reports [10,48]. To assess whether or not protein synthesis is required for the expression/maintenance of this form of LTD, we perfused the slices with cycloheximide (100 μM) throughout the experiment time-course. Fig 2B (red circles) shows measurements of fEPSP slope at 3 h, 6 h, and 8 h yielding values of 58 ± 1%, 68 ± 0.3%, and 69 ± 1%, respectively (*n* = 5), which are non-significantly different from LTD in cycloheximide-free slices.

**Fig 3:**
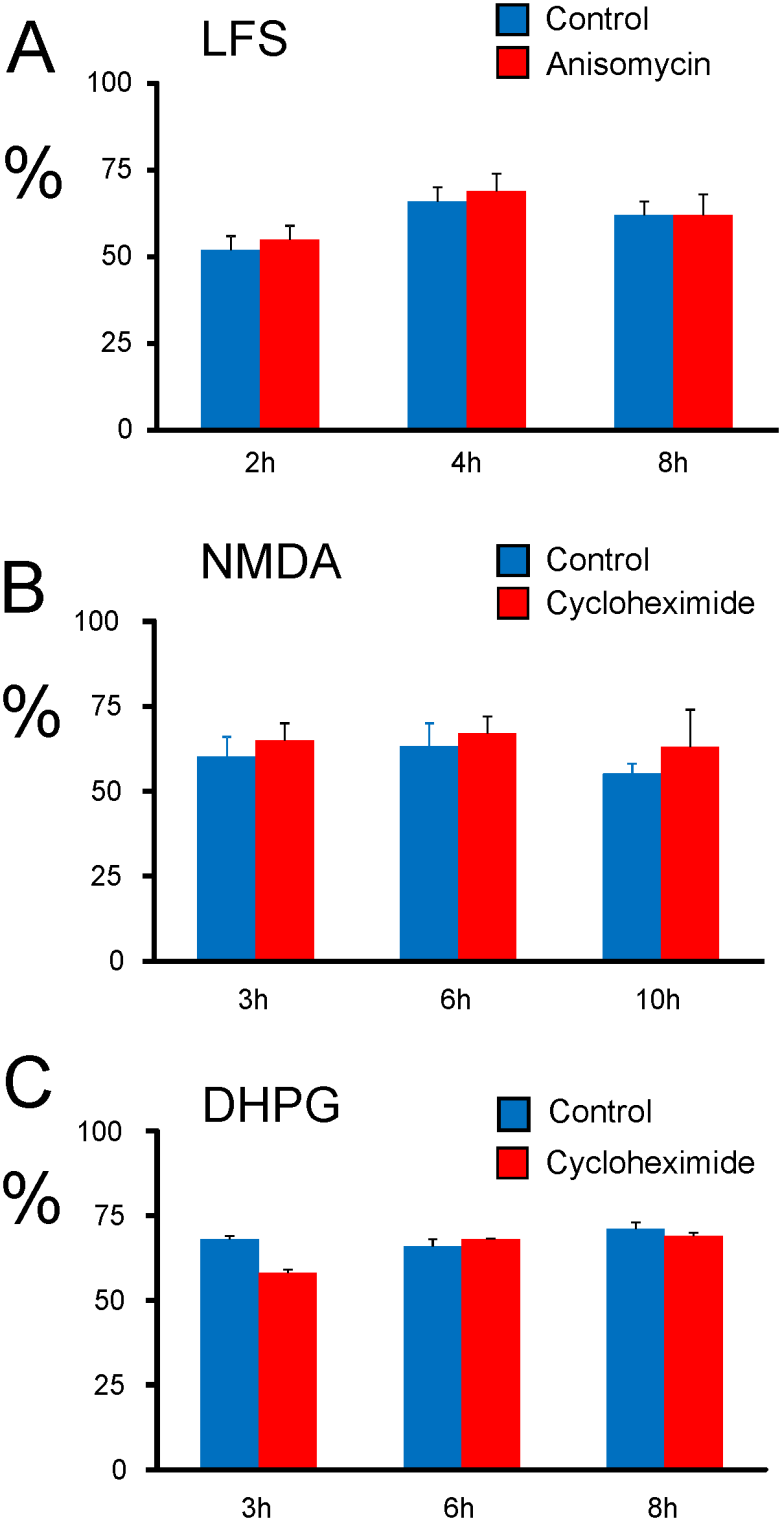
Quantification of LTDs with and without PSI. Cumulative histograms indicated LTD magnitude (mean ± SEM from groups of slices) after delivery of LFS or application of DHPG or NMDA alone (Control) or LFS, DHPG or NMDA plus PSI. Note also that the magnitudes and durations are nearly approximate indicating the generality of robustness. (A) Bar diagram showing LFS-LTD in slices treated with anisomycin vs. control slices. (B) Bar diagram showing NMDA-LTD in slices treated with cycloheximide vs. control slices. (C) Similar diagram but LTD was induced by DHPG. Both in LFS- NMDA- and DHPG-LTD, there was no significant effect when slices were incubated with PSI. The diagrams are based on the data in Figs 1 and 2. Results are depicted as mean ± SEM.

The comparisons depicted in Figs 1 and 2 are summarized in the bar plot diagram of Fig 3.

### NMDA-LTD might not be due to an NMDA-induced excitotoxicity

**Fig 4:**
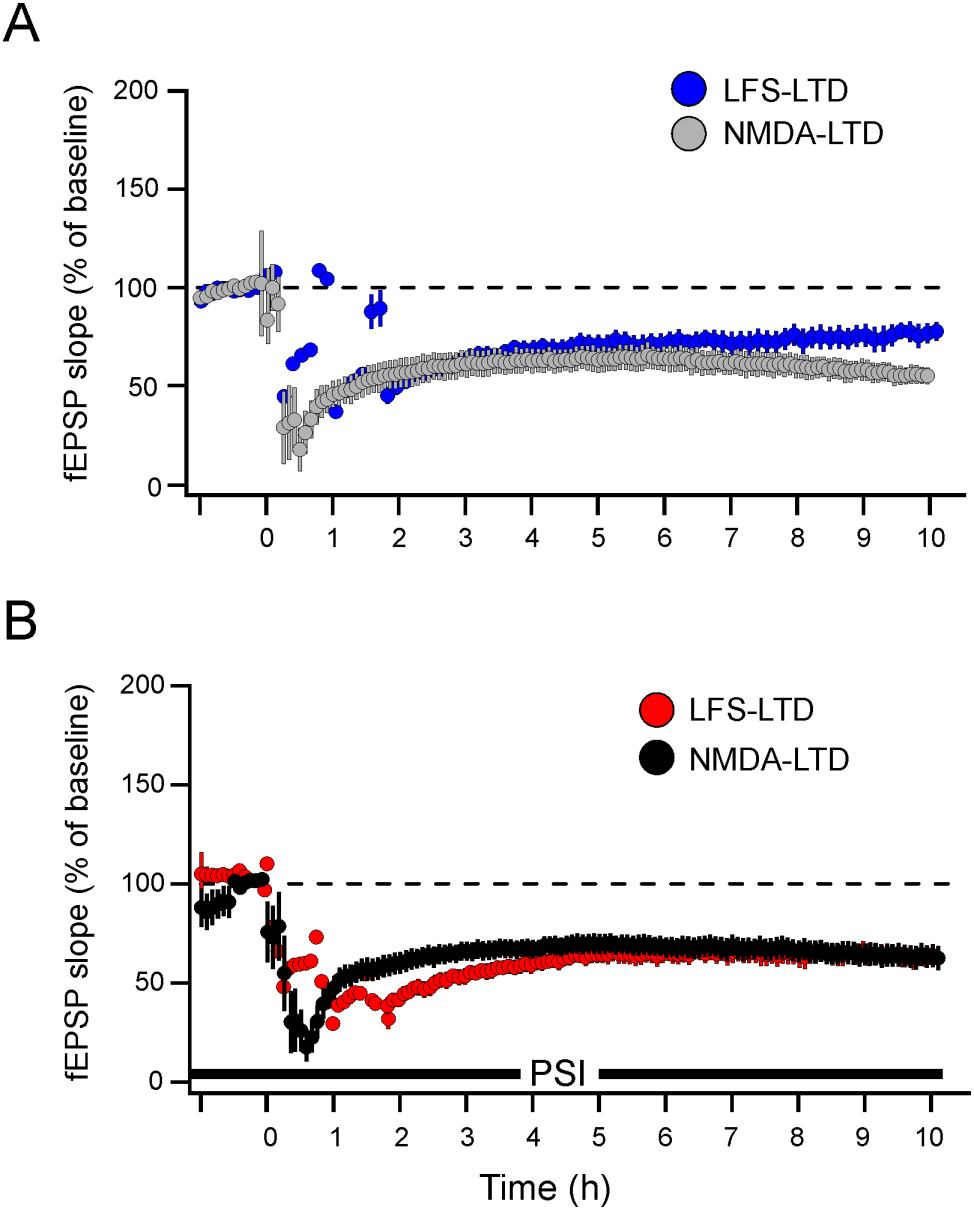
Comparisons of LTD magnitude in juvenile and middle-aged rats induced electrically or chemically, respectively. Superimposed data from Fig 1 on data derived from Fig 2A under control (A) and protein synthesis inhibition condition (B), indicates nonsignificant difference in magnitudes of LTD throughout the recording time courses.

NMDA is well established as neuronal excitotoxic, but at concentrations equal to or above than 50 μM and for application intervals equal to or longer than 5 min [49], the conditions that are not applied on the LTD induced by NMDA. Although the protocol used in this study was reported to induce chemical LTD rather than excitotoxicity [49], and that the magnitude of NMDA-LTD in the middle-aged rats is not significantly different from the magnitude of LFS-LTD in juvenile rats (Fig 4), the durable characteristic of the LTD still warrants a plausible apoptotic/excitotoxic underlying mechanism.

**Fig 5:**
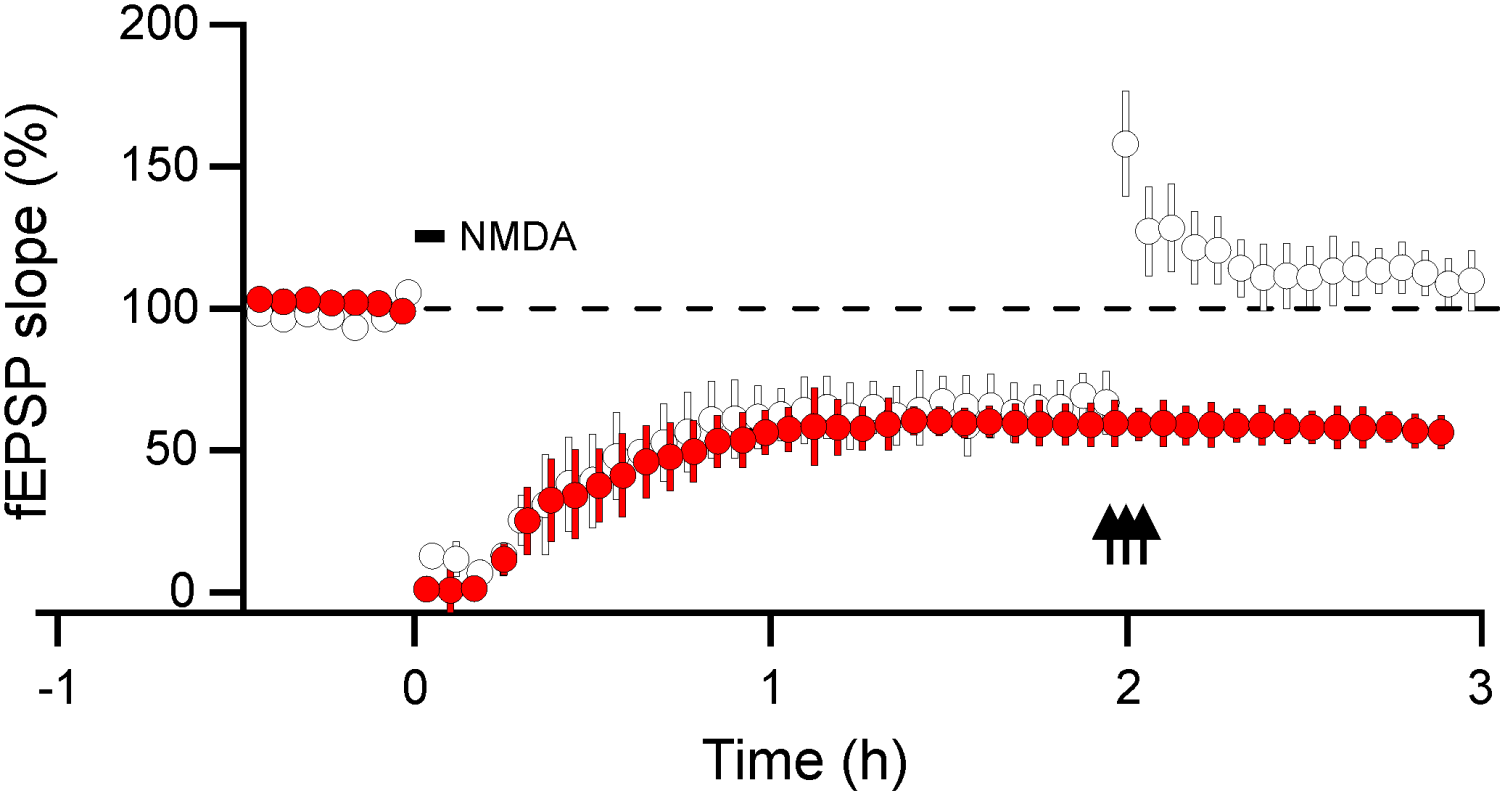
Reversal of NMDA-LTD by high-frequency stimulation. Three trains of tetanus (100 pulses at 100 Hz; triple arrow), spaced 5 s apart were delivered at about 2 h following NMDA (horizontal bar) washout in one input (open circles) caused homosynaptic recovery of the fEPSP to its pre-NMDA values (*n* = 6). Compare this result with the maintained LTD in the second input (red circles). Each represent the average of ~13 successive responses of *n* experiments and the amount of de-depression was expressed relative to pre-NMDA baseline values at 0 h.

We therefore conducted another set of experiments to verify or disprove such possibility by delivering three trains of tetanus (100 Hz, 5 s inter-train interval) to slices subjected to NMDA (and DMSO as vehicle) treatment to assess whether the NMDA-induced depression is reversible. From electrophysiological point of view, this parameter gives a clue for slice viability. As we unfortunately lacked more animals for this set of experiments, we used 16–20 days old rats. As Fig 5 shows, the magnitude of LTD induced by NMDA treatment in juvenile rats is comparative to that in the middle-aged rats. Tetanus trains delivered to one input 120 min after the establishment of NMDA-LTD were able to re-potentiate the depressed pathway. Although the recorded interval of the re-potentiation was relatively short, i.e. around 60 min, and the age of rats differs from the age used for NMDA-LTD, we suggest that the NMDA-induced depression preserved the slice for subsequent plasticity and that the NMDA-LTD might be reversible.

### DHPG caused an increase in the content of proteins

Although it has been shown that DHPG induces the synthesis of specific protein molecules [50,51], the drug has also been shown to induce global protein synthesis [23,52]. Consistently, mGluR-LTD is enhanced in the RNA binding proteins mutant mice such as the FMR protein [53] implying a role for general protein synthesis mechanisms. Accordingly, we estimated the change in the total amount of newly radiolabeled protein following DHPG treatment to hippocampal slices. As expected, DHPG induced a significant increase in the amount of total proteins compared to control slices S1C S1 (*p* < 0.05, one-sample *t*-test).

## Discussion

Two major forms of LTD triggered by the activation of NMDARs and mGluRs have been described in the central nervous system. These are induced either “physiologically” by trains of electrical stimulation [45] or can be mimicked by the application of specific agonists such as NMDA and DHPG [12,22,38–41]. It is canonically held that long-lasting synaptic plasticity is divided into an early phase, which are considered to be induced and expressed via non-translational mechanisms, and a late phase that is dependent on triggered *de novo* protein synthesis [14]. In previous works from our laboratory using juvenile rats, we found that LTP could be sustained for intervals bypassed the temporal limits of what is considered to be “E-LTP” under conditions of protein synthesis inhibition [28,44]. However, other groups demonstrated a possible stabilized protein synthesis-independent LTP in adult rodents [54–56]. However, it remains to examine whether these conflictual data can be applied on LTD, another form of synaptic plasticity, reported to contribute in some forms of hippocampal-dependent learning and memory [1,5,57–59].

In this report, distinct protocols induced distinct forms of robust and durable LTD in CA1 region were accomplished either electrically in slices obtained from juvenile rats or chemically in slices obtained from middle-aged rats, i.e. LFS-LTD vs. cLTD, or NMDA-LTD vs. DHPG-LTD. We have also tried to assess whether the durable NMDA-LTD was due to physiological or excitotoxic depression. Achieving “persistent” and “vigorous” LTD is a suitable tool for investigating whether or not *de novo* protein synthesis is necessary for LTD. We found that inhibition of protein synthesis by anisomycin or cycloheximide at reliable inhibitory concentrations and for long incubation interval did not interfere with the durability or magnitude of LTD. Through the chemical-induction procedure, DHPG maximizes synaptic depression, which is a favorable condition for detecting biochemical changes in the whole slice. Not surprisingly then that slices incubated with DHPG had higher protein contents than that found in slices incubated with aCSF alone. We propose that LFS-, NMDA- and DHPG-LTD can be stabilized for several hours in hippocampal slices without the requirement for triggered protein synthesis and regardless whether or not there was an increase in the newly synthesized proteins.

### Protein synthesis and LTD

There are several *in vitro* studies indicating that PSIs blocked the induction of LFS-LTD in juvenile [8] and young rats [9] as well as the induction of mGluR-LTD [9,10,17,32,35,36,39,47]. It has also been shown that the activation of mGluR-dependent signaling was tightly coupled to the regulation of translation in hippocampal neurons [60–62]. Nonetheless, despite it is found that DHPG caused an increase in the global protein synthesis, the present findings are yet contradictory to a majority of published works which considered a causative correlation between LTD and protein synthesis. These conflictual findings are located within the controversy whether or not protein synthesis has instructive role in hippocampal mGluR-LTD and NMDAR-LTD as under some circumstance they may not require protein synthesis. The current consensus posits favorable *de novo* protein synthesis-mediating processes for the LTD induced majorly by NMDA and more specifically by DHPG [27]. However, our present data and data shown by several other workers raise the possibility of two lines of reasoning. One implies no association between synaptic plasticity and acute protein synthesis. For example, while experiments in hippocampal slices originally suggested that rapamycin-mediated blockage of mammalian target of rapamycin complex 1 (mTORC1) impairs mGluR-LTD [39,40,52], recent findings have challenged this view [63], casting doubt on the contribution of mTORC1 to LTD. Moreover, Moult et al. [64] showed that LTD in 10–15 weeks old rats was not blocked by PSIs although the recorded interval was relatively short. On the other hand, Nosyreva and Huber [13] confirmed that the mGluR-LTD in neonatal rats (<2 weeks) is protein synthesis-independent, whereas it requires protein synthesis in adult rats (>3 weeks), although Zakharenko et al. [65] indicated a reverse finding in mice using LFS at 5 Hz frequency. However, in Nosyreva and Huber’s [13] study the recorded interval did not exceed 80 min in either age group. The other line of reasoning is that new protein synthesis might occur in LTD, as in any biological phenomenon but it has unlikely to have instructive role.

### How new protein synthesis may subserve LTD expression and maintenance?

Although the notion that LTP (and memories) requires an initial event of *de novo* protein synthesis seems reasonable, given it is suggested to be associated with an increase in the synaptic weights and efficacy, the notion that LTD also requires an immediate event of *de novo* protein synthesis is more problematic given it is suggested to be associated with a decrease in synaptic weights. However, there is some evidence supports the idea that *de novo* protein synthesis has a role in the regulation of ionotropic glutamate receptor trafficking and, in consequence, *de novo* protein synthesis is required ‘regardless of polarity’ [66] or regardless of gain and loss.

Although the induction of LTD is generally accomplished via an initial internalization of AMPA and NMDA receptors, the expression of LTD is debatable, which is presumed to be mediated by stabilization phase of those endocytosed receptors that renders LTD persistent [67–70]. As the internalized receptors to be persistently adhered to their intrasynaptic/extrasynaptic sites, it is this phase that is suggested to be dependent on protein translation [10,47,69,70]. This notion is based on the fact that the endocytosed glutamate receptors require protein molecules either to fix them in the intrasynaptic pool and/or to affix them with the extrasynaptic membrane [71]. Moreover, the removal of postsynaptic AMPAR and NMDARs may lead to structural changes, such as dendritic spine elongation, which is also initiated by mGluR-stimulated protein synthesis [66,72,73]. Although receptors internalization is also mediated by protein molecules [74–77], the issue why PSIs do not influence LTD induction has not been addressed despite the receptors recycling is over timescale that can range in minutes.

### DHPG and protein synthesis

Early studies have shown that glutamate triggered rapid increase in the size of polyribosome aggregates in synaptoneurosomes [27,51,78], and in transected dendrites [79,80] via mechanisms downstream the mGluRs activation.

In the present study, we examined hippocampal slices obtained from 24-day old rats, as at this age exaggerated mGluR- and protein synthesis-dependent LTD is observed [81]. As shown in S1C S1, there is a significant effect of DHPG on the total protein amount.

However, one may protest against the methodology of radiolabeled assay. The protocol for experiments shown in S1B and S1C Fig differs from the electrophysiological recording in terms of Ca^2+^ concentration, i.e. 2.5 mM vs. 1.5 mM, respectively. We indicate that this difference, as mentioned above, is based on age difference as the lower calcium concentration used in electrophysiological experiments was suitable for induction of LTD in middle-aged rats, the case that is not applicable on juvenile animals chosen for radiolabeled assay protocol.

### Constitutive proteins, its sources and protein breakdown

A puzzling issue remains to explain why and how the present findings are contradictory to the majority of published work and representing an anomaly to the dominant hypothesis that newly synthesized protein is necessary for the stabilization of synaptic plasticity. More puzzling though is that the experimental procedures used to perform the present findings were not radically unique from the ones used to study LTD although they did not mirror them exactly. As no definitive conclusion can be yet drawn from the present data, the point of departure to provide an explanation is that to disregard the canonical interpretive strategy of single working hypothesis usually inscribed in the positivist scientific tradition at the expense of multitude of hypotheses [82].

One suggestion might be that constitutive proteins, i.e. the protein repertoire, are under some conditions sufficient to assure LTD stabilization and, that posttranslational modifications (PTMs) are the determinant events [83]. In this regard, Auerbach and Bear [84] have shown that in case proteins are overexpressed, DHPG acts only via posttranslational mechanisms. Furthermore, neonatal rats were suggested to have sufficient protein levels to maintain mGluR-LTD in the absence of new synthesis [13]. The famous mice model proposed by Huber et al. [85] to imitate fragile X mental retardation syndrome revealed enhanced mGluR-LTD in CA1 that does not require new protein synthesis. In fact, the study implies the importance of overexpressed proteins over triggered protein synthesis. However, DHPG addition revealed an increase in protein contents in comparison to control slices S1C S1, indicating that DHPG-LTD is associated with new protein synthesis. However, the suggestion that constitutive proteins have determinant role does not radically diverge from new protein synthesis in case those proteins were dysfunctional/unfunctional as those proteins should be modified posttranslationaly before they are implicated in LTD stabilization.

Thus, the issue of constitutive proteins is necessarily coupled with another issue that is protein breakdown and protein half-lives, both are regulatable [86] and hence, sensitive to the experimental procedures (see next section). This is also applied on the newly synthesized proteins. In the first case, Ehlers [87] demonstrated that AMPARs undergo selective sorting between recycling and degradative pathways following activity-dependent endocytosis underlying a mechanistic link between chronic homeostatic synaptic plasticity, and synapse maintenance. However, as Rothman [88] states, “The complexities of the life history of proteins are enormous, as or more complex than the structure of these most complicated of molecules, and in some respects matches, perhaps unsurprisingly the complexity of life itself.” In the second case, the functionality of the newly synthesized proteins relevant to synaptic plasticity is determined by two major factors, the activity of enzymes such as kinases and phosphatases, and on the time-life of those molecules. Because of application of PSIs for several hours throughout the recording time did not influence LTD stability even though if LTD induction was associated with protein synthesis, there could be two alternatives: it can be presumed that triggered protein synthesis is necessary but the lack of effect of PSIs is attributed to “short” recording intervals (~8–10 h). Perhaps, long preincubation period of PSI before LTD induction, a la LTP [28] may exhaust the protein repertoire, which disrupts LTD because some of the biological phenomena are sensitive to even minute amount of protein depletion. Alternatively, the association of new protein synthesis to synaptic plasticity, in case the latter is a physiological phenomenon *par excellence* implies that the association is not essential for LTD stabilization that is, it has a permissive role. In either case, whether new protein synthesis does or does not occur would not change the fact that LTD, like LTP, is not dependent on new protein synthesis. If, on the other hand, one may presume that triggered protein synthesis is implicated on LTD stabilization via morphological change or other mechanisms but the effect of PSI would not be detected within 8–10 h recording interval, then the temporal dichotomy becomes superfluous and unnecessary.

In case the suggestion that constitutive proteins and the balance of synthesis/breakdown are crucial for synaptic plasticity stabilization (it is likely unnecessary to dichotomize synaptic plasticity for just to simulate memory dichotomy, itself problematic), one may wonder about the sources of these proteins. In addition to the well-documented evidence of a continuous movement of neurotransmitter receptors [89] and certain PSD scaffolding molecules [90,91] between intrasynaptic and extrasynaptic pools, which is strongly affected by LTD-inducing protocol [13,69,92], whether this process can sustain synaptic plasticity for longer duration or it subserves only the initial phase seems likely to be related to the identity of molecules, i.e. some molecules have dynamics of minutes time-scale while others have wider time-scales. Tsuriel et al. [93] have addressed this issue among several other issues. In cultured rat hippocampus neurons, the work revealed that the dynamics of molecules, such as synapsin, and ProSAP2 at individual central nervous system synapses may be dominated primarily by the continuous exchange and redistribution of synaptic proteins among nearby synapses lasting for several hours reaching to up 6 h, whereas protein synthesis and degradation may constitute slower, second-order processes that serve to maintain and regulate the size of local, shared pools of synaptic matrix proteins [93]. Although Tsuriel et al.’s [93] interpretation seems orthogonal to the proposal that it is the dynamics of protein synthesis/degradation that have a predictive power for the sensitivity or resistance to PSI treatment, we suggest that protein redistribution is the complementary to these dynamics (cf. [94]) in a way it may constitute a reserve that serves stabilization of biological phenomena against certain challenges and under specific experimental conditions such as the ones presented in this work.

### Why PSIs do not work in our experiments but not by others?

A conventional explanation could be attributed to methodological differences such as age difference (cf. [8–10,25]), species or strain difference (cf. [9–11,14,25]), type of preparation (cf. [8]), the type of incubation chamber (cf. [9]), incubation temperature (cf. [14]), adjusting the test stimulation strength (cf. [9,14]), and/or the levels of neuromodulators [95,96,97]. This suggestion by itself does not tell anything essential. Rather, what one may propose, more as a concluding gesture than a complete argument, is that an orchestration of the totality of experimental differences can tell how the balance or parity between protein synthesis and degradation might be of high significance. Achieving this goal requires a systematic assessment to elucidate the mechanisms by which the methodological variables interact with the intrinsic machinery that control protein dynamics. On the other hand, the present data raises the problem of reproducibility, central to scientific hypothesis building. There are two aspects that deserve to pay attention. The first one is whether or not there is variability from experiment to experiment. If there was such variability, the quite accepted methodology is to discard those results that do not match the premise, usually considered as noise, outliers, or behavioral deviations from the predicted outcomes. This would lead to, borrowing from the psychological terminology, an implicit bias. In consequence, the reproducibility is misused although it remains essential for the scientific inquiry. Second, that is more challenging is whether or not there is variability from laboratory to laboratory. The history of biology discipline tells us that there is no phenomenon that has never been disputed. Indeed, the failure to reproduce findings shown by other laboratories is not *per se* the problem given the complexity of the biological phenomena on the one hand, and the proliferation of inquirers groups, each has its own premises, predictions, experimental procedures, statistical methods, criteria for data selection, interpretations, and experimentally verified hypotheses, on the other hand. A trial that may resolve these problems could be a methodology such like established on the evidence-based medicine, that is meta-analysis and critical appraisal, which call us for evidence-based neurobiology.

## Supporting Information

S1 Fig Diagrammatic representation of radiolabeled assay and the effect of DHPG on protein amounts. ‘(A) A circulation system mimicking electrophysiological procedures with exception of no electrical stimulation. The incubation chamber is a modified 8-well dish whereby oxygenated aCSF circulates via separate inlets for each well and two outlets collecting the flowing aCSF from a hole made in each well. One side of the chamber (4-well) contained hippocampal slices perfused with drug-free, DMSO-containing aCSF (control) whereas the other 4-well side contained slices perfused with DHPG/D-APV-containing aCSF. The flowed aCSF was not recirculated as it was a spilled mixture of drug-containing and drug-free perfusates. Arrows indicate the direction of aCSF flow. (B) Representation of schedules for experiments of DHPG effect on protein turnover. Hippocampal slices were first incubated at room temperature with oxygenated aCSF for 60 min, mimicking preincubation of slices used for electrophysiological experiments. Then, slices were transferred to incubation chamber (A) and equilibrated with circulating aCSF (31°C) for further 20–30 min before they were treated either with DMSO-aCSF (control) or DHPG/D-APV-aCSF for 10 min. Washout interval (20–30 min) preceded a 50-min tritiated leucine incubation, which was followed by termination and incorporation assay. (C) The effect of DHPG on the newly synthesized protein. Histogram shows mean protein quantity (% of control; dashed line) estimated via leucine incorporation immediately following DHPG washout. Application of 100 μM DHPG caused significant increase in total protein amount (*n* = 5; *p* < 0.005). Data are represented as mean ± SEM.
(TIF)

## Abbreviations

aCSF: artificial cerebrospinal fluid
ANI: anisomycin
cLTD: chemical-LTD
D-APV: D-(-)-2-amino-5-phosphonopentanoic acid
DHPG: (*RS*)-3,5-dihydroxyphenylglycine
fEPSP: field excitatory postsynaptic potential
FMR: fragile mental retardation protein
LTD: long-term depression
LTP: long-term potentiation
LFS: low-frequency stimulation
mGluR: metabotropic glutamate receptor
NMDA: N-methyl-D-aspartate
NMDAR: NMDA receptor
PSI: protein synthesis inhibitor
