# Supplementary figures and images for: Protein Synthesis Inhibitors Did Not Interfere with Long-Term Depression Induced either Electrically in Juvenile Rats or Chemically in Middle-Aged Rats
Source: PLoS One. 2016 Aug 12;11(8):e0161270. doi: 10.1371/journal.pone.0161270 (PMC4982604; doi:10.1371/journal.pone.0161270)

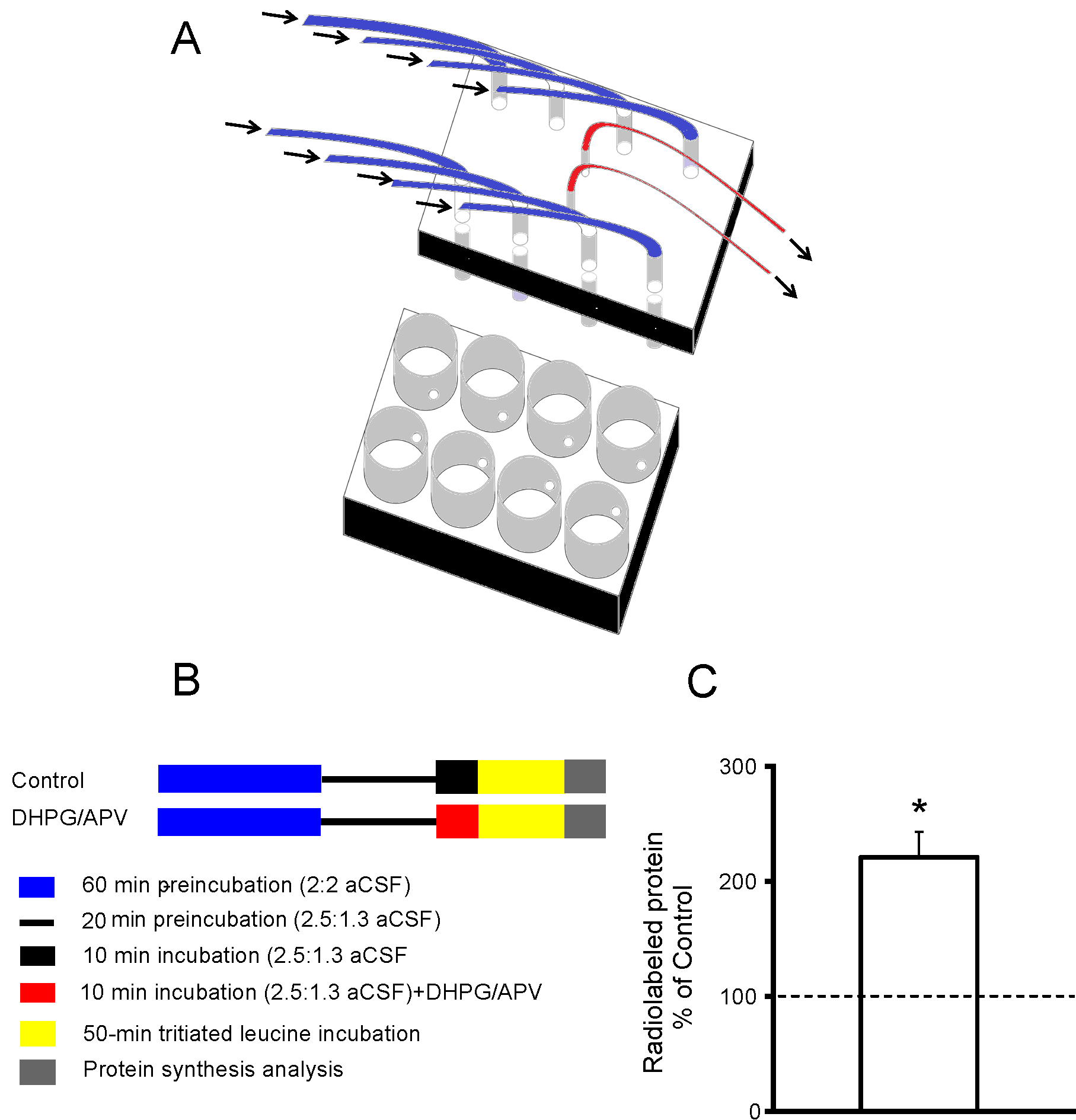

Supplement: S1 Fig — ‘(A) A circulation system mimicking electrophysiological procedures with exception of no electrical stimulation. The incubation chamber is a modified 8-well dish whereby oxygenated aCSF circulates via separate inlets for each well and two outlets collecting the flowing aCSF from a hole made in each well. One side of the chamber (4-well) contained hippocampal slices perfused with drug-free, DMSO-containing aCSF (control) whereas the other 4-well side contained slices perfused with DHPG/D-APV-containing aCSF. The flowed aCSF was not recirculated as it was a spilled mixture of drug-containing and drug-free perfusates. Arrows indicate the direction of aCSF flow. (B) Representation of schedules for experiments of DHPG effect on protein turnover. Hippocampal slices were first incubated at room temperature with oxygenated aCSF for 60 min, mimicking preincubation of slices used for electrophysiological experiments. Then, slices were transferred to incubation chamber (A) and equilibrated with circulating aCSF (31°C) for further 20–30 min before they were treated either with DMSO-aCSF (control) or DHPG/D-APV-aCSF for 10 min. Washout interval (20–30 min) preceded a 50-min tritiated leucine incubation, which was followed by termination and incorporation assay. (C) The effect of DHPG on the newly synthesized protein. Histogram shows mean protein quantity (% of control; dashed line) estimated via leucine incorporation immediately following DHPG washout. Application of 100 μM DHPG caused significant increase in total protein amount (n = 5; p < 0.005). Data are represented as mean ± SEM. (TIF) [file pone.0161270.s001.tif]
